# STED nanoscopy: a glimpse into the future

**DOI:** 10.1007/s00441-015-2146-3

**Published:** 2015-03-06

**Authors:** Paolo Bianchini, Chiara Peres, Michele Oneto, Silvia Galiani, Giuseppe Vicidomini, Alberto Diaspro

**Affiliations:** 1 Nanoscopy, Nanophysics, Istituto Italiano di Tecnologia, Via Morego 30, 16163 Genova, Italy; 2 Present Address: MRC Human Immunology Unit and Wolfson Imaging Centre Oxford, Weatherall Institute of Molecular Medicine, University of Oxford, Oxford, UK

**Keywords:** Super-resolution microscopy, Fluorescence, Stimulated emission

## Abstract

The well-known saying of “Seeing is believing” became even more apt in biology when stimulated emission depletion (STED) nanoscopy was introduced in 1994 by the Nobel laureate S. Hell and coworkers. We presently stand at a juncture. Nanoscopy represented a revolution in fluorescence microscopy but now is a mature technique applied to many branches of biology, physics, chemistry, and materials science. We are currently looking ahead to the next generation of optical nanoscopes, to the new key player that will arise in the forthcoming years. This article gives an overview of the various cutting-edge implementations of STED nanoscopy and tries to shine a light into the future: imaging everything faster with unprecedented sensitivity and label-free.

## Introduction

Conventional fluorescence microscopy is decisive for addressing central challenges in many areas of life science, but the diffraction phenomenon defines a limit to the achievable spatial resolution. The diffraction limit is usually quantified by Abbe’s law. This law states that the ability to distinguish two objects separated by distance *d* depends on the wavelength *λ*, the refractive index of the medium *n*, and the focusing power of the lens sin*θ* used to observe the objects, i.e., *d = λ/2*(*n*sin*θ*), where *n*sin*θ* corresponds to the numerical aperture (NA) of the objective lens (Abbe 1873). In visible and near-infrared (NIR) fluorescence microscopy, this means, in practice, that the structures can be resolved if they lie apart by more than 200–300 nm. Until 1994, optical microscopy was widely believed to have reached such a limit. However, in that year, Hell and Wichmann (1994) introduced the stimulated emission depletion (STED) concept as a method to break the diffraction barrier. That seed has now matured into a technique that has been developed in several different directions; it allows unprecedented new possibilities for the investigation of the structure and function of sub-cellular components. In 2014, its clear impact on physics, biology, and chemistry was endorsed by the Nobel Prize (Betzig et al. 2014). In order to overcome the diffraction barrier, Hell and co-workers implemented the idea of squeezing the effective fluorescence volume of a scanning microscope by a process called stimulated emission (SE). The fluorophores located at the periphery of the excited region can be quenched by a second beam, the so-called STED beam, which features a zero intensity point at the center. The most typical configuration of the STED beam is a doughnut-shape intensity distribution. The STED beam stimulates the emission of the fluorophores, instantaneously bringing them to the ground state. The wavelength of the depletion beam should be red-shifted to the tail of the fluorophore emission spectrum. In order to obtain unlimited resolution, however, the stimulated emission process should saturate and consequently broaden the effective doughnut area of depletion, and hence, the resolution now relies only on the power of the applied STED beam. Therefore, the spontaneous fluorescence emission will occur only in the very center of the excited volume from a region that will become smaller and smaller as the power increases (Klar et al. 2000; Hell et al. 2004; Harke et al. 2008). Thus, the final resolution, *d*, of the microscope will be well described by the following relationship: *d = λ/*(*2NA* $\sqrt{1+I/{I}_S}$), where *I* is the maximum intensity of the STED beam, and the saturation intensity *I*_*S*_ is the intensity required by the STED beam to quench the spontaneous fluorescence emission by half (Westphal et al. 2008). Since the described process together with the scanning of the sample is immediate, STED nanoscopy allows the direct acquisition of super-resolved images and, in general, does not require any further computational post-processing. The STED concept has been generalized to any systems in which light can switch the molecule between two states (Dyba et al. 2003; Hell 2007). The idea applies to techniques such as ground state depletion (GSD; Hell and Kroug 1995) and reversible saturable optical fluorescence transitions (RESOLFT; Hofmann et al. 2005; Grotjohann et al. 2011). Both these methods rely on dark and bright states, but light emission is not a limiting factor; for instance, in nanolithography, the states involved are polymerizing and non-polymerizing states (Harke et al. 2013). Another highly interesting variant is based on a pump–probe process whereby a pump perturbation of charge carrier density in a sample and the consequent change in transmission of the probe are the key elements for super-resolution (Silien et al. 2012; Wang et al. 2013). Although these last-mentioned techniques will be some of the main actors in future developments, we focus our attention here on the well-established STED nanoscopy technique.

## Typical STED

**Fig. 1:**
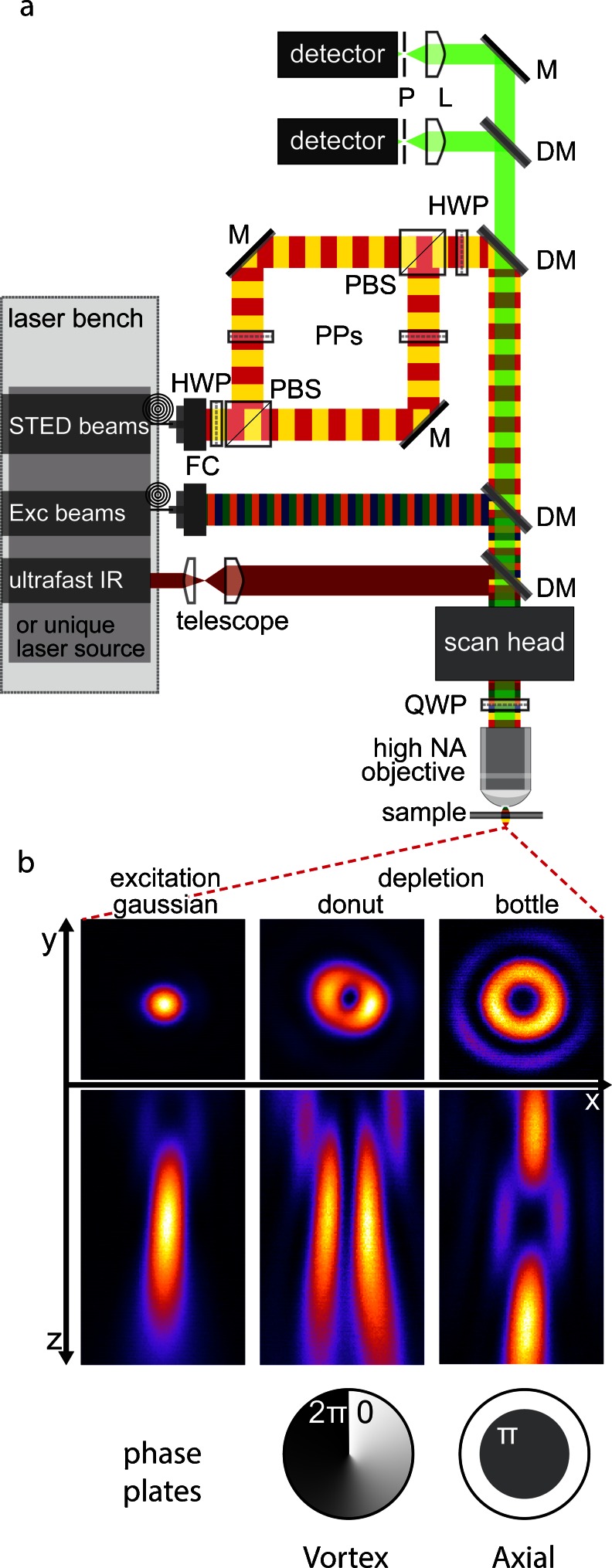
**a** Generalized stimulated emission depletion (*STED*) set-up. We show several laser beams in order to take into account the multicolor STED set-ups and to emphasize the huge laser availability and the large possible combinations that allow full customization of the architecture (*Exc* excitatory, *IR* infrared, *NA* numerical aperture, *HWP* half wave plate, *QWP* quarter wave plate, *PBS* polarizing beam splitter, *PPs* phase plates, *M* mirror, *DM* dichroic mirror, *L* lens, *P* pinhole). **b** Beam profiles as they appear at the focal plane and the respective phase plates that generate them

A typical STED nanoscope is similar to a confocal microscope. It needs at least two co-aligned laser beams: one for excitation and a second for the depletion of fluorescence (Fig. 1). The spatial profile of the STED beam has to feature a zero intensity point at the center. To achieve an annular pattern along the lateral plane, the most commonly used approach is the introduction of a vortex phase plate (Keller et al. 2007). If resolution improvement is required along the optical axis, then the right choice is a bottle profile made by an axial phase plate (Fig. 1b; Klar et al. 2000). However, in general, a combination of both profiles is preferable. This guarantees more flexibility, allowing a custom three-dimensional nanoscale resolution as the result of the experiment. As depicted in Fig. 1a, a polarizing beam splitter divides the STED beam into the two paths that correspond to the two phase plates; this ensures the possibility of balancing the power of the two profiles and, hence, of adjusting the overall three-dimensional resolution on demand (Harke et al. 2008).

## Multicolor STED

**Fig. 2:**
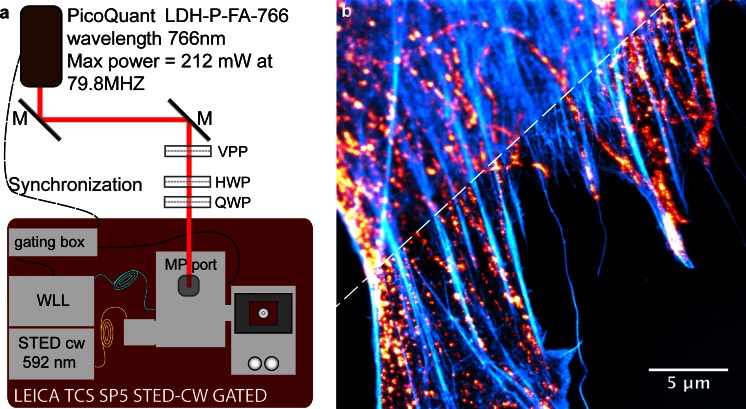
Customized commercial STED set-up and example of the image that it can acquire. **a** Representation of a set-up that is essentially constituted by a commercial Leica TCS SP5 gated STED-CW whereby a powerful and triggerable pulsed laser at 766 nm (Picoquant LDH-P-FA-766; Picoquant, Berlin, Germany) has been coupled through the Leica Multi-Photon port, the beam profile has been made into a doughnut-shape by a vortex phase plate, and the pulses have been electronically synchronized with the supercontinuum laser source used in the Leica microscope for excitation. In this configuration, we combine two different STED approaches, namely the gCW-STED with pulsed STED implementation. Such a set-up allows multicolor STED nanoscopy with green and far-red emitting fluorescent molecules. **b** Example of confocal (*top left*) and STED (*bottom right*) multicolor images acquired with the set-up shown in **a**. The sample is a 3T3 cell in which we have labeled actin filaments with Alexa Fluor 488 phalloidin (*cyan*) and tubulin with Abberior Star 635p (*red*). The image has been collected sequentially by two Hybrid detectors in the spectral range 500–550 nm and 650–720 nm, respectively. We have used an HCX PL APO CS 100x 1.4NA oil objective (Leica Microsystems, Mannheim, Germany) and a scan speed of 1400 Hz for 2048 pixel per line with a 64-line average. Confocal resolution (*top left*) shows apparent filaments for actin and tubulin. The STED image (*bottom right*) reveals that the tubulin filaments are not continuous and actually feature fairly well-separated spots. This is a typical labeling artifact that becomes evident in super-resolution microscopy and should be avoided

Although even one color STED made by a pair of co-aligned beams can be considered a good achievement, currently, multicolor STED imaging is feasible. For instance, in a set-up equipped with one STED beam, two-color STED nanoscopy can be achieved by adding a second cheap excitation beam and by selecting the fluorophores such that they have different excitation spectra but a similar emission tail, and thus, the same depletion beam can be used for both (Schmidt et al. 2008). Another approach suitable for performing two-color STED is the combination of two pairs of excitation and depletion beams (Figs. 1a, 2). In these cases, we should choose each fluorophore so that it works optimally for each pair of beams (Donnert et al. 2007; Meyer et al. 2008; Blom et al. 2011). Three-color STED, although more difficult, has been achieved by the use of two pairs of laser beams and the discrimination of the fluorophores on the basis of their lifetime and their spectral properties (Bueckers et al. 2011). A more convenient and promising method is the use of the newly developed large Stokes-shift dyes, e.g., Abberior STAR 470SX (Abberior, Göttingen, Germany) or ATTO 490LS (Atto-Tech, Siegen, Germany), which allow the separation of the third color (or the second) by the excitation wavelength.

## Pulsed STED

**Fig. 3:**
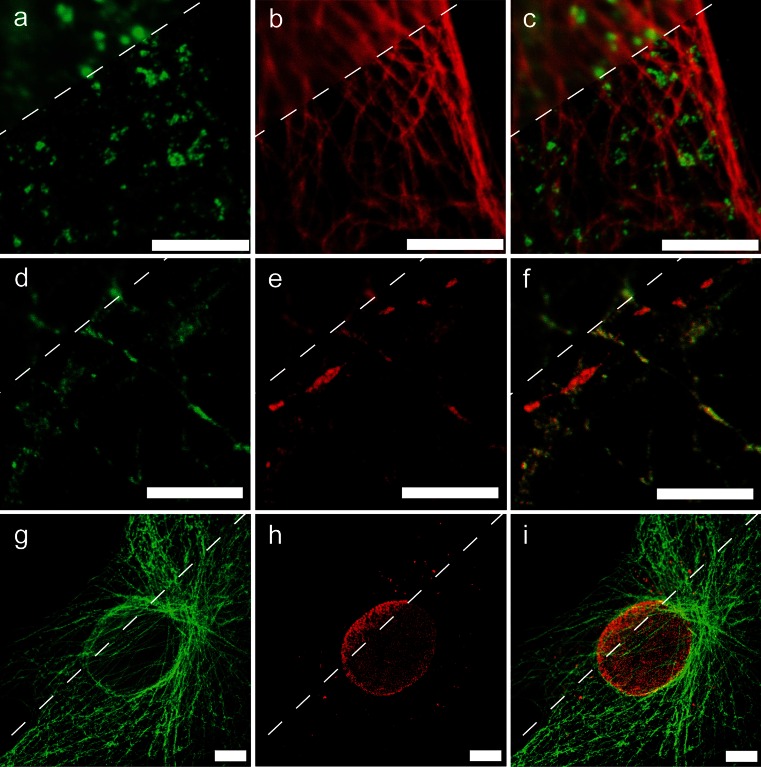
Three examples of multicolor STED imaging. **a–f** Images were acquired by a custom-made pulsed STED microscope based on a supercontinuum laser source (Galiani et al. 2012b). **g–i** Images were acquired by a standard commercial Leica TCS SP5 gated STED-CW microscope (Leica Microsystems, Mannheim, Germany). *Top left corners* Confocal image. *Bottom right corners* STED image. **a–c** Multicolor imaging of Hela cells in which clathrin is stained by ATTO 590 (*green*) and tubulin by ATTO647N (*red*). **d–f** Culture of hippocampal neurons in which SynI is labeled with ATTO 594 (*green*) and vGAT with Abberior STAR635p (*red*). Sample courtesy of Marta D’Orlando and Gabriele Lignani, Neuroscience and Brain Technologies, Istituto Italiano di Tecnologia, Genova, Italy. **g–i** Ptk2-cell in which microtubules are labeled by Alexa488 (*green*) and NUP153 by Abberior 440SX (*red*). **a**, **d** Excitation wavelength is 566 nm, STED wavelength is 715 nm, and collection spectral window is 570–640 nm. **b**, **e** Excitation wavelength is 640 nm, STED wavelength is 745 nm, and the collection spectral window is 650–690 nm. **g**, **h** Excitation wavelength is 470 and 488 nm, respectively, STED wavelength is 592 nm for both, the collection spectral window is 480–500 nm and 510–560 nm, respectively. **c**, **f**, **i** Color overlay of the respective images *left*. *Bars* 5 μm

Hell and coworkers built their first STED set-up by using synchronized trains of pulses for excitation and depletion (Klar et al. 2000). The main reasons were the need for a high flux of stimulating photons within the short (1–5 ns) excited-state lifetime of the fluorophores to quench effectively the fluorophore in the focal periphery and thus improve the spatial resolution of the system. In this configuration, a depletion pulse immediately follows the excitation pulse. In general, the excitation pulse should be much shorter than the STED pulse, while the depletion pulse should be longer than the vibrational relaxation time (>50 ps), but shorter than the fluorescence lifetime of the fluorophore (<250 ps). A complete study of the effects of various pulses is provided by Leutenegger et al. (2010). Nevertheless, the use of longer STED pulses is not prohibited; merely, some early spontaneous photons can skip the pulse, and so, a time-gating detection approach is necessary to bring the resolution performance of the nanoscope to its maximum (Galiani et al. 2012b). A pulsed STED architecture is commonly considered expensive; the main reason is probably that, at the time of the first implementation, the preferred laser sources were a combination of a Ti:Sapphire laser and an optic parametric oscillator (OPO), which were the most obvious sources able to satisfy the power and wavelength requirements for STED (Willig et al. 2006a, 2006b). Nowadays, some alternative laser sources present a cheaper and easier solution. Among these, we include stimulated Raman scattering fiber laser sources (Rankin and Hell 2009) and super-continuum laser sources (Wildanger et al. 2008, 2009; Galiani et al. 2012a) such as the Fianium ALP-710-745-SC (Fianium, Southampton, UK), which was used to acquire the first two rows of images in Fig. 3. In this last case, the laser delivers both the excitation and the depletion beams, and the train of pulses are synchronized, but the fine temporal tuning has to be made optically. Notably, the recently introduced pulsed triggerable high-power laser diodes from Picoquant (Berlin, Germany; Fig. 2) allow electronic synchronization to any pulsed laser source used for excitation. The use of such a laser offers similar facility as the continuous wave (CW) lasers in the case of CW-STED.

Among the advantages of pulsed STED is the relatively low average power requirement for depletion. Nevertheless, the power has to be two orders of magnitude larger than the excitation power, but not as high as for multiphoton excitation (Diaspro et al. 2005). Another advantage that can be achieved by using a low (a few MHz or less) repetition rate is that the fluorophores are allowed to relax from triplet states before being re-excited (Donnert et al. 2006), and hence, photobleaching is reduced.

## STED by CW lasers and time-gated approach

An important innovation towards the wide-dissemination of STED microscopy was the demonstration of the use of continuous-wave (CW) lasers as the STED beam (Willig et al. 2007). The possibility of producing the STED beam with a CW laser reduces complexity (no pulse elaboration is needed) and costs and increases the versatility of the system: CW lasers provide almost any wavelengths in the visible and near-infrared region. However, since the peak intensity supplied by a STED beam running in CW is lower with respect to a pulsed beam, the resolution performance of a CW-STED system lags behind that of a pulsed STED system. A solution to this problem is obtained by combining CW-STED microscopy with a time-gated detection scheme, the so-called gated CW-STED (gCW-STED) implementation. With a CW-STED microscope, one can easily demonstrate that the relative probability of silencing a fluorophore increases if the fluorescence is collected after a time-delay from the excitation event of the fluorophore. The reason is that the inhibition of fluorescence by stimulated emission strongly depends on the number of stimulating photons to which the fluorophore is exposed while residing in the excited state. If the fluorescence photons are collected only after a time > *T*_g_ from the excitation events of the fluorophore, then this ensures that the collected fluorescence stems from fluorophores that have resided in the excited-state for at least a time > *T*_g_ and have thereby been exposed to stimulating photons for at least the same amount of time. In practice, the fluorophores are excited all at the same time through a pulsed excitation laser, and the time-gated detection allows early fluorescence photons to be discarded (<*T*_g_). As a result, the ability to quench fluorophores for a given STED laser power substantially improves. In the particular context of imaging, the longer the time-delay *T*_g_ between the excitation and the fluorescence collection, the more one ensures that the fluorescence is recorded mainly from fluorophores located in the doughnut center in which the STED beam intensity is “zero”, and thus, the higher is the effective spatial resolution. Unfortunately, the reduction of the effective collection volume comes together with a reduction of the overall signal that forms the image (Vicidomini et al. 2013b). Thereby, the signal-to-noise (SNR) and the signal-to-background ratio reduction impose an upper limit in the choice of the time-delay *T*_g_. Different hardware- and software-based approaches have been employed to push this upper limit. In particular, different lock-in and synchronous detection schemes have been combined with gCW-STED to remove uncorrelated background sources, such as the anti-Stokes fluorescence emission potentially induced by the STED beam itself (Vicidomini et al. 2012; Coto Hernández et al. 2014). Furthermore, ad-hoc deconvolution algorithms, able also to reassign the early photons, instead of simply rejecting them, have been implemented and have demonstrated a significant recovery of the SNR with respect to the raw gated STED images (Castello et al. 2014).

## Two-photon excitation STED

Two-photon excitation (2PE) microscopy is a well-known technique for the study of thick biological tissue. Although it relies on a non-linear quadratic dependence of the excitation power, 2PE does not improve the spatial resolution per se. As it was first demonstrated in 2009 (Moneron and Hell 2009; Ding et al. 2009), 2PE fluorescence microscopy could be readily combined with STED approaches allowing super-resolution imaging of mouse brain (Bethge et al. 2013). 2PE microscopy normally adopts a pulsed ultrafast Ti:Sapphire laser source for the excitation beam, and thus, when combined with a pulsed STED beam, it needs expensive and sophisticated instrumentation able to synchronize the two lasers. Nevertheless, it probably offers several advantages in terms of performance (Takasaki et al. 2013). The best-balanced approach is the use of CW lasers for depletion. This method, as previously mentioned, does not require synchronization and guarantees good resolution that can be directly improved by time-gated detection (Vicidomini et al. 2013a). Notably, novel and cheaper pulsed 2PE-STED has been demonstrated (Bianchini et al. 2011). Such an approach exploits, for STED, the same Ti:Sapphire laser as that used for 2PE. The main requirement is for a fluorophore that can be excited and depleted at the very same wavelength, e.g., ATTO647n (Atto-Tech, Siegen, Germany).

Such a single wavelength 2PE-STED technique has the advantages that no other lasers are necessary for STED, it is a pulsed implementation, and it is easy to achieve. However, the excitation wavelength cannot be optimal for the fluorophore, hence reducing SNR.

## STED for the study of molecular dynamics

Fluorescence correlation spectroscopy (FCS) represents an established method for studyng the motion of single molecules and its binding properties in solution, cells and tissues. Moreover, scanning microscopy imaging has been applied to add a spatial dimension to the classic FCS modality: spatiotemporal FCS (stFCS) measures the time and the routes that the diffusing particles or molecules follow to go from one place to another in the specimen (Digman and Gratton 2011). STED nanoscopy has been recently combined with all these FCS techniques, i.e., STED-FCS (Eggeling et al. 2009), raster imaging correlation spectroscopy (RICS) STED (Hedde et al. 2013), and cross-pair correlation spectroscopy (pCF) STED (Bianchini et al. 2014).

## Correlative microscopy

Fluorescence microscopy and nanoscopy are still unique in offering highly specific, three-dimensional imaging of living systems (Diaspro et al. 1996; Osseforth et al. 2014). However, electron microscopy (EM) and near field scanning probe techniques, such as atomic force microscopy (AFM), might offer complementary information and bridge the still existing resolution gaps. In particular, correlative microscopy (Vicidomini et al. 2010) is of primary interest, because it allows the observation of the cell/structure/event by two complementary techniques, e.g., STED nanoscopy and EM (Watanabe et al. 2011), or STED nanoscopy and AFM (Harke et al. 2012). Moreover, the advantage of a correlative approach is not only in terms of pure imaging, but also in terms of additional chemical and mechanical information about the sample. Eventually, one of the combined techniques might become an active factor, for example, by manipulating the specimen by AFM while imaging by STED (Chacko et al. 2014).

## Concluding remarks

The application of STED nanoscopy to biology often requires a shift in thinking. At first glance, one can imagine that the STED method generates a better and new view of biological structures by making visible aspects hidden by conventional fluorescence microscopy. However, actually, what we now see also represents a large amount of data that needs to be carefully interpreted in order to exploit fully the power of the STED technique.

The ultimate resolution in STED nanoscopy, as in other super-resolution techniques, is the single fluorescent molecule. Thus, special care should be directed to the preparation of the sample and, in particular, the immunostaining. The use of primary and fluorescently labeled secondary antibodies results in an apparent broadening of the organelle under study, thereby affecting the interpretation of the spatial distribution of the labeled protein. Furthermore, since the size of the antibody construct might hamper the recognition of contiguous targeted proteins, label density might be deficient (see Fig. 2b; Mueller et al. 2011). Nevertheless, the advantage of using this labeling method is the excellent fluorescence intensity that can be achieved which fluorescently labeled primary antibody, for instance, cannot always guarantee. In any case, for quantitative studies, e.g., correlation, colocalization, or distribution analyses, labeling procedures should be carefully evaluated in order to avoid systematic errors in the study of the targeted protein position. However, labeling procedures are now standardized, and the literature contains many examples of quantitative STED imaging in which immunostaining has been successfully employed (Willig et al. 2006b; Kittel et al. 2006). Good examples of immunostaining in living systems, mainly in neuroscience applications, are also available (Westphal et al. 2008). Nevertheless, STED can be used with fluorescent proteins such as green or yellow fluorescent protein (Willig et al. 2006a; Sieber et al. 2007; Bianchini et al. 2014) or snap-tag fusion proteins (Hein et al. 2010).

Sample requirements, good fluorophore availability, and laser convenience were, for many years, some of the limiting factors for building one's own custom system and hence for broadening the use of STED nanoscopy. However, today, Leica Microsystems (Mannheim, Germany), Abberior Instruments (Göttingen, Germany), and Picoquant (Berlin, Germany) offer easy-to-use and flexible commercial STED nanoscopes. Moreover, many components of a STED nanoscope are now cheaper and easy to buy, even making simpler the achievement of custom-made STED nanoscopes. This will probably mean that STED nanoscopy will become a routinely used technique in many labs and microscopy facilities.

The future challenges remain the usual ones: the attainment of new horizons in terms of speed, depth, and color multiplicity, while maintaining resolution at the molecular level in a living biological specimen.
